# Transformation of CLL to ALCL: the role of clonality studies in diagnostic molecular haematopathology

**DOI:** 10.1007/s12308-016-0280-9

**Published:** 2016-08-08

**Authors:** Richard Colling, Daniel Royston, Elizabeth Soilleux

**Affiliations:** 1 Department of Cellular Pathology, Oxford University Hospitals NHS Trust, Oxford, UK; 2 Department of Oncology, University of Oxford, Oxford, UK; 3 Nuffield Division of Clinical Laboratory Sciences, University of Oxford, Oxford, UK

**Keywords:** Lymphoma, PCR, Clonality, Molecular diagnostics

## Abstract

Clonality studies greatly assist in the diagnosis of challenging haematopathology cases. These robust and standardised tests aid the detection of clonal lymphoid populations and may assist in lymphocyte subtyping. In this case report, a gentleman presented with a high-grade transformation of a B cell neoplasm which histologically and immunophenotypically mimicked a T cell anaplastic large-cell lymphoma. With the aid of T cell and B cell receptor clonality studies, it was demonstrated that this tumour was in fact of B cell lineage. This report exemplifies the role of these increasingly used and relatively new molecular tests in unusual and difficult lymphoma presentations and highlights potential pitfalls in the interpretation of their results.

## Introduction

The diagnosis and subtyping of lymphoproliferative disorders is increasingly aided by evaluating the repertoire of B (Ig) and T cell (TCR) receptors within lymphoid populations [1, 2]. Ig and TCR are antigen recognition molecules on the surface of lymphocytes which are encoded by unique V(D)J exons formed by somatic recombination of *V* (variable), *D* (determining) and *J* (joining) gene regions during early maturation in primary lymphoid tissues [3]. Characterising V(D)J regions present in a lymphoid population not only is extremely useful in distinguishing reactive (polyclonal) from malignant (clonal) processes but aids in subtyping lymphocytes with aberrant immunophenotypes [1, 4, 5]. This report aims to demonstrate the clinical utility of clonality studies in an unusual case and highlights potential pitfalls when interpreting molecular data.

## Clinical history

**Fig. 1:**
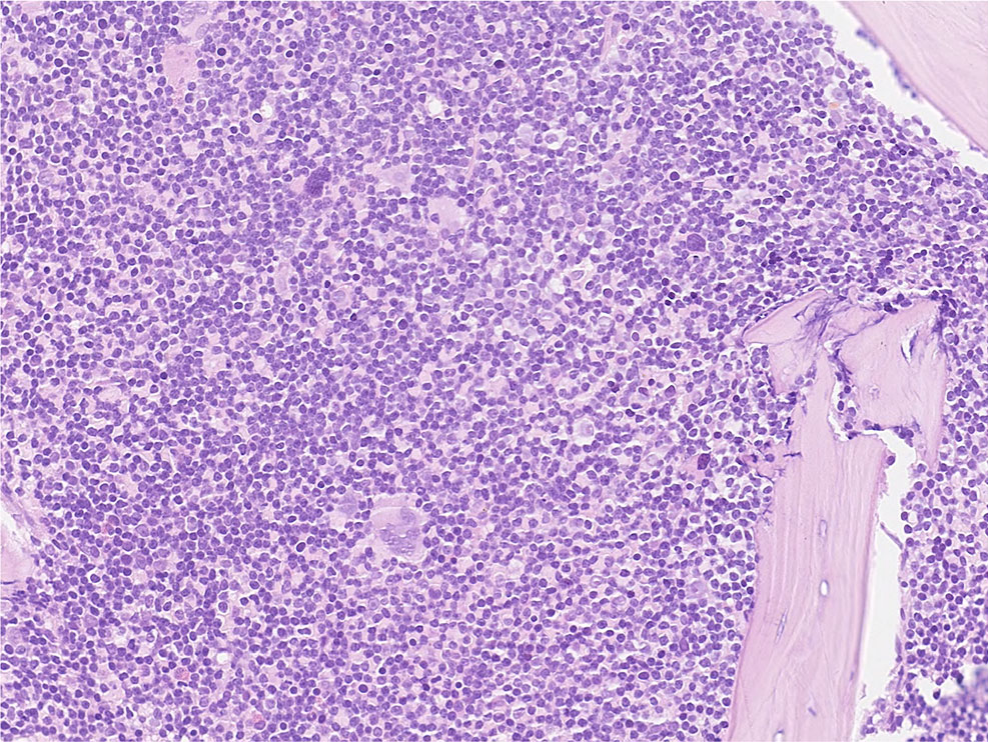
Histology of the bone marrow trephine biopsy showing a dense infiltrate of small monomorphic lymphoid cells. These cells were immunopositive for CD5, CD23 and BCL-2 but immunonegative for CD10 and cyclin-D1, in keeping with involvement by CLL (H&E; ×425 magnification)

A 43-year-old gentleman with no remarkable medical history presented in 2005 with a lesion of the right buccal mucosa. This was biopsied and reported as non-specific inflammation. Eight years later, he presented with an enlarged right-sided neck lymph node which underwent core biopsy. The histology of this revealed a dense infiltrate of monomorphic lymphoid cells which were immunopositive for CD5, CD23 and BCL-2 but immunonegative for CD10 and cyclin-D1. A diagnosis of a B cell neoplasm, small lymphocytic lymphoma (SLL)/chronic lymphocytic leukaemia (CLL) was made. A review of the previous buccal biopsies confirmed a diagnosis of CLL in these also. Watchful waiting was pursued. One year later, he presented with a recurrence of CLL at his previous neck site and underwent bone marrow trephine, which demonstrated infiltration by CLL, as shown in Fig. 1. The gentleman remained well with conservative treatment until around a year later when he re-presented with anaemia, multiple buccal lesions and an extensive left scrotal mass.

**Fig. 2:**
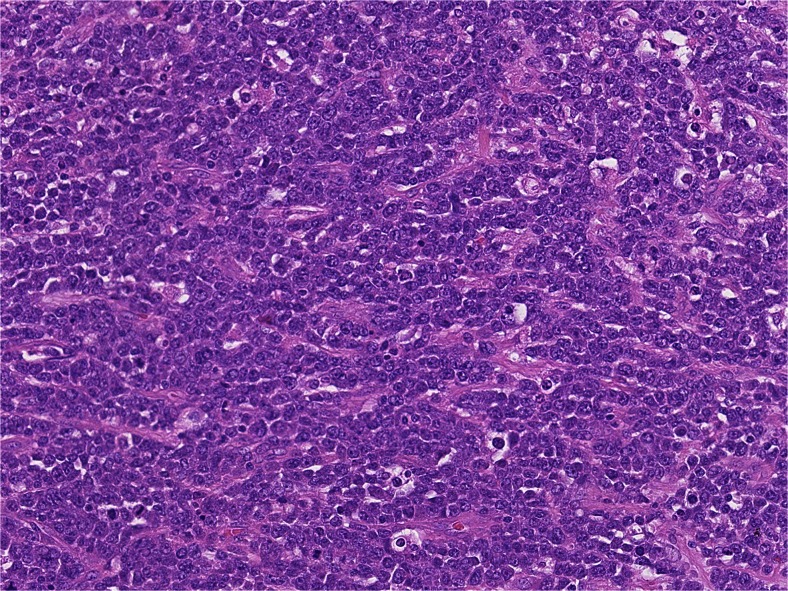
Histology of the left scrotal resection showing a dense infiltrate of large, high-grade lymphoid cells. These cells were immunopositive for CD30 and CD7 and immunonegative for ALK1, in keeping with ALK-negative ALCL (H&E; ×425 magnification)

The gentleman underwent an incisional biopsy of a buccal lesion and a left orchidectomy with scrotal skin resection. Histological examination of both showed an extensive infiltrate of large, high-grade lymphoid cells with the morphological characteristics of anaplastic large-cell lymphoma (ALCL), as shown in Fig. 2.

## Materials and methods

IHC and in situ hybridization (ISH) was initially performed to subtype the high-grade tumour. B cell receptor (BCR) and T cell receptor (TCR) clonality studies were carried out due to the unusual presentation. Clonality studies were performed using the polymerase chain reaction (PCR)-based IdentiClone Gene Clonality Assays (Invivoscribe Technologies, Inc.) [6] which are EuroClonality validated multiplex protocols.

## Results

The left testis was extensively infiltrated by lymphoma, extending into the scrotal soft tissues and overlying skin. The malignant cells demonstrated immunopositivity staining for CD30 and CD7. The Ki67 proliferation fraction was almost 90 %, and anti-c-myc IHC showed staining in around 80 % of the cells. There was variable weakly positive immunostaining for CD79a, CD2, CD4, CD8, TIA-1 and BCL-6. IHC for CD20, CD19, CD5, CD10, CD56, TdT, CD68 (PGM1 clone) and ALK1 was negative. Chromogenic ISH staining for Epstein-Barr virus (EBV) was negative. The buccal infiltrate showed similar morphological and immunophenotypical appearances, with additional IHC stains showing CD45 positivity and CD3, Pax5, CD68 (KP1 clone), lysozyme, CD34, cytokeratin and Melan A negativity.

**Fig. 3:**
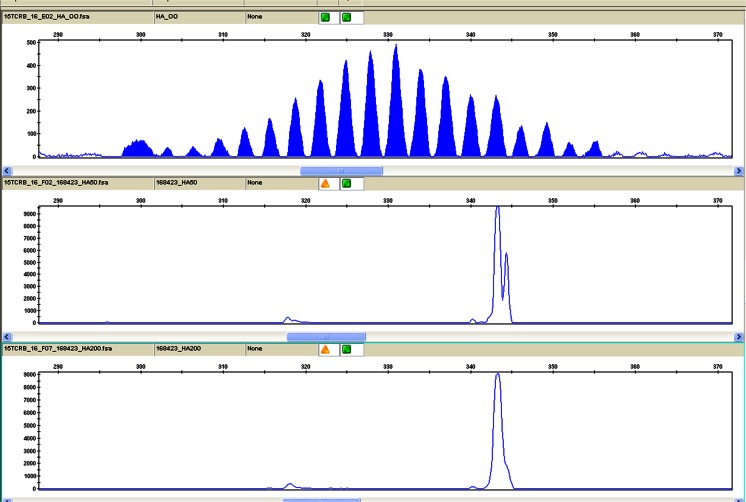
IdentiClone Gene Clonality Assay results from the buccal tumour showing the PCR GeneScan traces for the Ig heavy chain V_H_-J_H_ gene rearrangements (size range 310–360 nt). There is a large clonal peak at 343n, reproducible at both 50 ng (*middle trace*) and 200 ng (*bottom trace*) concentrations compared with the polyclonal control (*top trace*). The pseudo-double peak appearance at 343–344 base pairs in the PCRs produced using 200 ng of DNA template is due to the large amount of PCR product at 343 base pairs, saturating the electrophoretic capillary system during GeneScanning, giving an appearance of a second peak with slower electrophoretic migration (344 base pairs). Similar clonal peaks were also demonstrated with Ig heavy chain V_H_-J_H_ gene rearrangement reaction mixes at size ranges 250–295 and 100–170 nt, kappa Vk-K_de_ (size ranges 120–160, 190–210 and 260–300 nt) and kappa Vk-K_de_+intron K_de_ (size ranges 210–250, 270–300 and 350–390 nt) reaction mixes and were also present in the same reaction mixes from the buccal tissue tested

**Fig. 4:**
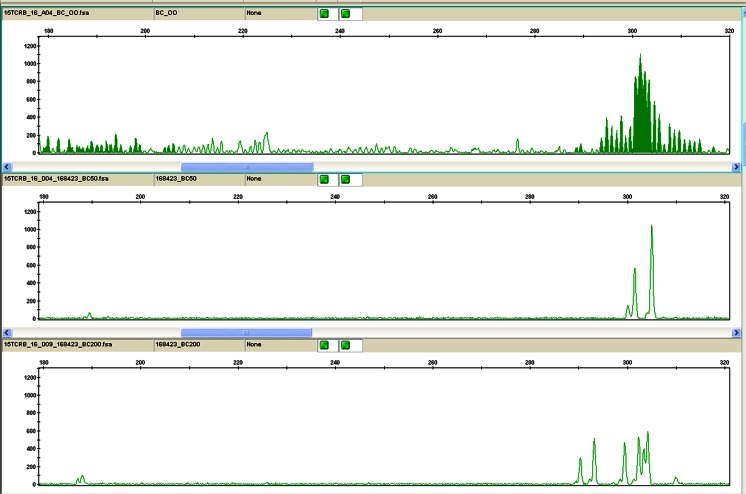
IdentiClone Gene Clonality Assay results from the buccal tumour showing the PCR GeneScan traces for the TCR Dβ1+Jβ1 (size range 285–325 nt) and Dβ2+Jβ1 (size range 170–210 nt) gene segments. The Dβ1+Jβ1 segment shows a restricted and irregular polyclonal profile in both 50 ng (*middle trace*) and 200 ng (*bottom trace*) concentrations when compared with the polyclonal control (*top trace*). The Dβ2+Jβ1 segment shows a dominant peak at the 50 ng concentration. This is not reproducible at 200 ng, but instead shows a restricted and irregular oligoclonal profile at this concentration. This suggests that there is a scanty background population of T cells with an oligoclonal (likely reactive) response, producing a pseudoclonal peak at the low concentration, due to the low numbers of T cells. Similar findings were present in several of the other TCR reaction mixes in both the testicular and the buccal tissue. Several T cell reaction mixes failed to amplify, presumably due to insufficient T cell derived DNA

Multiplexed PCR amplification of extracted DNA from both the testis and buccal mucosa produced a large isolated peak with all Ig heavy chain and kappa reaction mixes, as shown in the example in Fig. 3. These results demonstrated a clonal rearrangement of both loci within the malignant lymphoid population, consistent with a B cell origin. Amplification of TCR loci performed on the testis, however, produced a somewhat restricted and irregular profile with inconsistent fragment sizes between replicates, as shown in Fig. 4. A number of similar irregular peaks were also identified in some of the TCR rearrangement results. There was insufficient material to perform clonality studies on the antecedent CLL, and the patient died shortly after the diagnosis of ALCL.

## Discussion

ALCL is a high-grade lymphoma characterised by large, pleomorphic CD30 positive cells typically with a cytotoxic T cell phenotype [7]. ALCL derived from B cell precursors has been described and possibly lies within the spectrum of diffuse large B cell lymphoma (DLBCL) [5, 7–9]. Transformation of CLL to a high-grade tumour, known as Richter’s transformation, occurs in around 5–10 % of SSL/CLL. Most commonly transformation is to DLBCL; however, Hodgkin lymphoma, composite lymphomas and dendritic cell sarcomas have been reported. Transformation to an ALCL is unusual [10–12]. Although the tumour shares an anaplastic morphology with that of T cell ALCL, the two entities should be considered distinct in terms of pathogenesis and prognosis. Overlapping morphology and immunophenotype, however, make the distinction challenging. Therefore, clonality studies can be extremely useful in this setting [9, 13]. In particular, in a case such as this, it is useful to determine whether the ALCL represents a transformation of the underlying CLL or is a second independent lymphoma.

Clonality was evaluated historically with labour-intensive and error-prone Southern blotting. In recent years, PCR-based assays have gained popularity [1, 2, 4, 14–18]. PCR product size is dependent upon random assortment during recombination, and when visualised with GeneScan analysis software (Applied Biosystems) [19], a reactive population shows a Gaussian curve distribution [1, 14]. In contrast, neoplastic populations result in a single peak [1, 14]. The selection of assay primer targets for a case depends on the clinical question as well as the quality and quantity of DNA extracted; however, standardised multiplex assay protocols have been developed and validated by the EuroClonality consortium (formally BIOMED-2) and these have resulted in highly sensitive and specific tests which are now considered standard practice. These are commercially available to all diagnostic molecular pathology centres [1, 4, 14, 15, 17, 18, 20].

In this case, the gentleman developed a high-grade lymphoid malignancy following an indolent 10-year history of CLL. The patient then rapidly deteriorated and died shortly after surgery. The initial morphological and immunophenotypical impression was that of a de novo ALCL with a T cell phenotype, raising a possibility of dual pathology [5, 21, 22]. This would be extremely unusual and, therefore, PCR-based clonality studies were performed to aid the diagnosis. This convincingly demonstrated that the population was of B cell origin, in keeping with CLL which had undergone transformation to ALCL and gained an aberrant T cell phenotype [1, 5]. Interestingly, a number of small irregular peaks in TCR amplicons were also present, and this could be misinterpreted as confirming a T cell neoplasm with background CLL. However, partial rearrangement of T cell V(D)J regions within B cell neoplasms is well recognised [23–25]. Oligoclonal T cell proliferation in immune responses to lymphoproliferative disorders is also well documented [1, 26, 27], and DNA from these scanty populations, diluted into the background of tumour and normal tissue, can mimic malignancy with pseudoclonal peaks at low DNA concentrations and suspicious oligoclonal profiles at higher concentrations. These are potential pitfalls when not considered within the full clinical context [4, 13, 28].

## Take-home messages

- Lymphoproliferative disorders with overlapping morphological and immunophenotypical features continue to pose a challenge for diagnostic histopathologists.
- PCR-based molecular clonality studies with EuroClonality endorsed assays are being increasingly used to aid in such diagnoses.
- These studies reveal diagnostically useful information about clonality in lymphocyte populations and can be useful in subtyping B and T cells.
- Interpretation of these studies must be carried out with caution and within the full clinical and morphological context in order to avoid pitfalls.
